# Fatal Case of *Naegleria fowleri* Primary Amebic Meningoencephalitis from Indoor Surfing Center, Taiwan, 2023

**DOI:** 10.3201/eid3009.231604

**Published:** 2024-09

**Authors:** Hsin-Yi Wei, Yi-Wen Lai, Shu-Ying Li, Yen-I Lee, Meng-Kai Hu, Da-Der Ji, Chia-ping Su

**Affiliations:** Taiwan Centers for Disease Control, Ministry of Health and Welfare, Taipei, Taiwan (H.-Y. Wei, Y.-W. Lai, S.-Y. Li, Y.-I. Lee, M.-K. Hu);; National Yang Ming Chiao Tung University, Taipei (D.-D. Ji);; New Taipei Municipal Tucheng Hospital and National Tsing Hua University, Hsinchu, Taiwan (C.-p. Su)

**Keywords:** *Naegleria fowleri*, primary amoebic meningoencephalitis, meningitis/encephalitis, parasites, next-generation sequencing, syndromic surveillance, surfing, indoor recreational waters, Taiwan

## Abstract

We investigated a fatal case of primary amoebic meningoencephalitis from an indoor surfing center in Taiwan. The case was detected through encephalitis syndromic surveillance. Of 56 environmental specimens, 1 was positive for *Naegleria fowleri* ameba. This report emphasizes the risk for *N. fowleri* infection from inadequately disinfected recreational waters, even indoors.

Primary amoebic meningoencephalitis (PAM) is a rare but fatal disease caused by *Naegleria fowleri*, a single-cell, free-living ameba. *N. fowleri* amebae usually live in natural waters, but occasionally can be found in swimming pools, surf parks, and other recreational waters that have not been adequately disinfected (*1*). We report a case of PAM in Taiwan from an indoor recreational water facility.

## The Study

A 30-year-old woman visited an indoor surfing center in northern Taiwan in July 2023. Five days after her visit, she started experiencing neurologic symptoms, including headache and stiffness in her shoulders and neck. Fever and generalized tonic-clonic seizures then occurred, and she was admitted to the hospital. Her condition deteriorated rapidly, and she died 3 days later. The hospital reported the case to the Taiwan Centers for Disease Control (TCDC) as encephalitis with an unknown cause.

In 2010, TCDC implemented encephalitis syndromic surveillance to identify the causative agents responsible for cases of unexplained encephalitis (*2*). Surveillance involves screening for 41 pathogens by using multiplex quantitative reverse transcription PCR (qRT-PCR) (*2*). In this case, *N. fowleri* was the only pathogen detected from the patient. After next-generation sequencing analyses, the TCDC reference laboratory assembled the complete 49,558-bp *N. fowleri* mitochondrial sequence from the patient (*3*) and submitted the sequence to GenBank (accession no. OR459835).

The investigation team and the local public health authority (LPHA) went to the surfing center to conduct an environmental investigation and took various water and biofilm samples to test for *N. fowleri* (Table; Appendix). TCDC classified this work as a public health investigation; thus, it was not subject to institutional review board approval.

**Table:** Environmental samples collected and qRT-PCR results in an investigation of a fatal case of *Naegleria fowleri* primary amebic meningoencephalitis from indoor surfing center, Taiwan, 2023*

Sample collection site	No. samples	Results
Water samples		
Swimming pool	5	Negative
Spa pool	5	Negative
Reservoir of surf pool	13	Negative
Two inflatable outdoor swimming pools	6	Negative
Stagnant water under basement ladder	1	Positive, Ct 33.55
Biofilm samples		
Water inlet of swimming pool	1	Negative
Main drains of swimming pool	2	Negative
Connection outlet of swimming and spa pools	1	Negative
Water outlets of spa pool	2	Negative
Water inlet of spa pool	1	Negative
Main drain of spa pool	1	Negative
Showerheads of spa pool	1	Negative
Surf ramp	5	Negative
Reservoir of surf pool	12	Negative

*Ct, cycle threshold; qRT-PCR, quantitative reverse transcription PCR.

**Figure 1:**
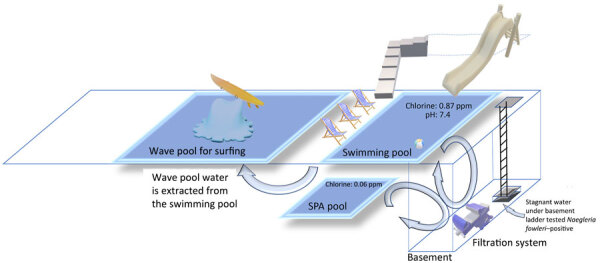
Illustration of 3 pools and basement mechanical area in an investigation of a fatal case of *Naegleria fowleri* primary amebic meningoencephalitis from indoor surfing center, Taiwan, 2023. Chlorine levels for pools are shown. Water for the wave pool was extracted from the swimming pool, and no additional chlorine was added to the wave pool. ppm, parts per million.

**Figure 2:**
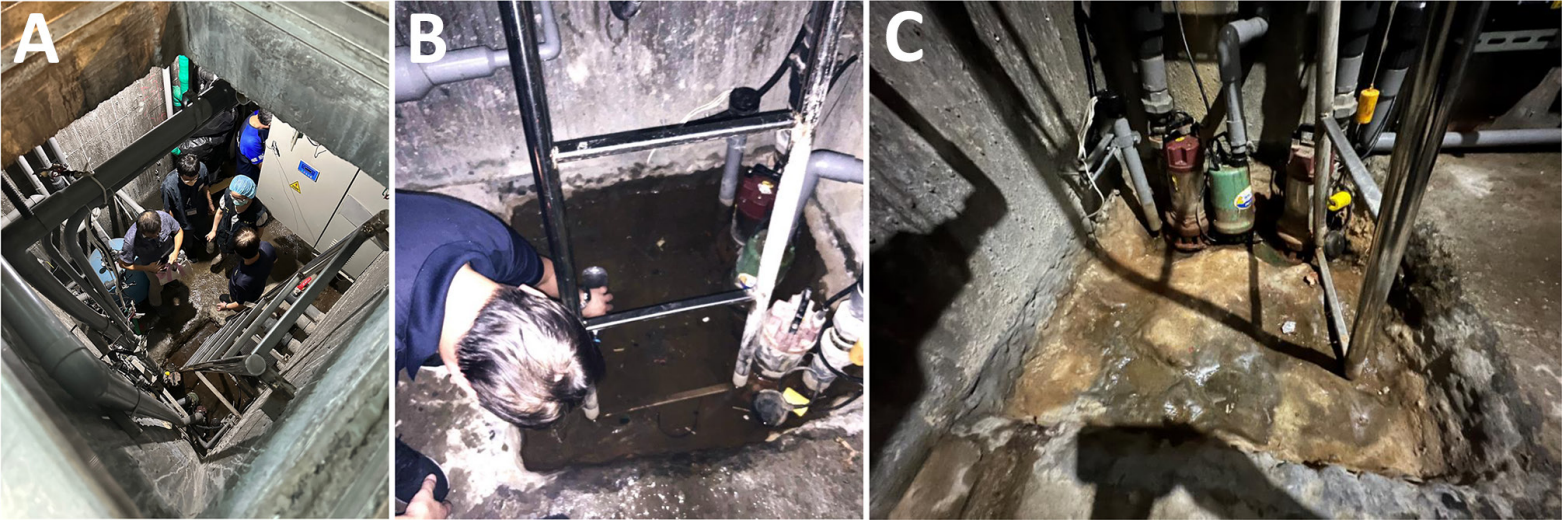
Basement mechanical area in an investigation of a fatal case of *Naegleria fowleri* primary amebic meningoencephalitis from indoor surfing center, Taiwan, 2023. A) The opening to the basement with ladder visible to right of opening; B) stagnant water beneath the basement ladder; C) depression under basement ladder after water was drained.

The surfing facility includes a swimming pool, spa pool, wave pool, dining area, and shower rooms on the first floor (Figure 1). A waterslide, gym, and billiards are on the second floor, and a basketball court is on the third floor. The basement is equipped with a water filtration system that has a chlorination function and can only be accessed by ladder (Figure 2, panel A). The water supply is municipal tap water. The water for the wave pool is extracted from the swimming pool, not directly from the municipal water source. The facility drains water from the pools every 2­–3 months for thorough pool cleaning. Staff members add chlorine powder after the facility closes each day to maintain the chlorine level in the pool, but they do not add chlorine to the wave pool. Staff conduct water quality tests every day but do not keep records of results. 

Before this fatal case occurred, the pool had not been completely drained and refilled for >2 months. However, before the investigation team took samples, most of the water had been drained from the pools and the pools had been cleaned.

After the onsite investigation, water quality testing of collected specimens revealed the following: chlorine content was 0.87 parts per million (ppm) in the swimming pool and 0.06 ppm in the spa pool; pH value was 7.4 in the swimming pool and unavailable in the spa pool (Figure 1). The normal residual chlorine standard for swimming pools is 1–3 ppm; thus, the test results for the collected specimens revealed the chlorine content was insufficient.

We collected a total of 30 water samples and 26 biofilm swab samples, which we submitted for qRT-PCR testing (Table). Among those specimens, 55 from the pool were negative, but 1 specimen from stagnant water beneath the basement ladder was positive for *N. fowleri* (Figure 2). That water might have been seepage from pipes associated with the pump motor, but further investigation showed that the positive specimen was not associated with pool water. Because employees access the basement daily to operate the filtration system, we could not rule out the possibility of pool contamination from staff shoes or feet.

The surf center had several design deficiencies, such as overlapping pathways connecting each facility. The staircase leading from the swimming pool to the second-floor water slide is used by visitors to general activity areas, such as the gym or basketball court. The facility did not provide a footbath for swimmers to use before entering the pool, and the pool had no drainage ditch along its edges. Such design deficiencies might increase the risk for external contamination to be introduced to the swimming pool.

The LPHA traced 12 employees and 630 customers by telephone, but none reported symptoms during the surveillance period. The surfing center suspended operations for comprehensive cleanup and disinfection. The facility addressed deficiencies and completed renovations before it reopened. The staff members were instructed to maintain free chlorine levels at 1–3 ppm and a pH of 7.2–7.8. 

## Conclusions

PAM cases are rare. In the United States, 0–5 cases were diagnosed annually during 2013–2022 (*1*). In some instances, *N. fowleri* has been found in underchlorinated recreational waters. However, the event we describe occurred in an indoor water body that was entirely supplied by municipal tap water. Although we found 1 positive environmental sample, the nucleic acid within the specimen was insufficient for sequencing and could not be used to prove a connection between the case and the *N. fowleri* in the environment.

The negative results from other samples might be related to the fact that most of the pool water had been drained and the pool had been cleaned before we conducted environmental sampling. The stagnant water that yielded *N. fowleri–*positive qRT-PCR results was collected from the damp, unpainted basement (Figure 2). That water might have been seepage from pipes, but the ameba could have originated from cracks in the floor or walls or from dirt on the shoes of staff members going into the basement. Unfortunately, we did not gather other environmental specimens from the basement, nor did we sample the shoe soles of staff members working in the basement. Consequently, the origin of *N. fowleri* and its transmission pathways in this case remained unknown.

Activities known to pose a higher risk for *N. fowleri* infection include diving and jumping into water. For beginner surfers, maintaining balance on the water can be difficult, and falling into the waves frequently enables water to flow into the nose. In 2018, a man in New Jersey, USA, also died from PAM after visiting a surf resort (*4*). We recommend that persons playing in recreational waters avoid letting water enter the nasal cavity.

Chlorine decay might be more challenging to address in wave pools for surfing because such levels can be higher in turbulent water surging at high speeds (*5*,*6*). However, current guidance on chlorine maintenance specific to wave pools is relatively lacking. In the surfing center we investigated, the water in the wave pool is extracted from the swimming pool. The staff adds chlorine powder to the swimming pool but not to the wave pool. Therefore, chlorine concentration could be even lower in the wave pool than in the swimming pool, which already had insufficient chlorine levels. Because we did not isolate the free-living ameba from the positive sample, we could not conduct a chlorination sensitivity test.

In conclusion, insufficient levels of residual chlorine can pose a risk for PAM, even in indoor water facilities. Indoor facilities should emphasize water quality and sanitation. Facility owners should maintain daily disinfection practices and LPHA should conduct inspections to ensure water quality in recreational facilities. 

Appendix Additional information on fatal case of *Naegleria fowleri* primary amebic meningoencephalitis from indoor surfing center, Taiwan, 2023.
