# Supplementary material for: Fatal Case of Naegleria fowleri Primary Amebic Meningoencephalitis from Indoor Surfing Center, Taiwan, 2023
Source: Emerg Infect Dis. 2024 Sep;30(9):1922–5. doi: 10.3201/eid3009.231604 (PMC11346987; doi:10.3201/eid3009.231604)
Supplement: Appendix — Additional information on fatal case of Naegleria fowleri primary amebic meningoencephalitis from indoor surfing center, Taiwan, 2023. [file 23-1604-Techapp-s1.pdf]

Article DOI: https://doi.org/10.3201/eid3009.231604

EID cannot ensure accessibility for supplementary materials supplied by authors. Readers who have difficulty accessing supplementary content should contact the authors for assistance.

Fatal Case of *Naegleria fowleri* Primary Amebic Meningoencephalitis from Indoor Surfing Center, Taiwan, 2023

Appendix

Encephalitis Syndromic Surveillance

Laboratory Methods

The targets screened by multiplex qRT-PCR include 41 pathogens: 36 viruses, 2 bacteria, and 3 protozoa (*1*): *Toxoplasma gondii, Naegleria fowleri,* and *Acanthamoeba* spp. To enhance the accuracy of qRT-PCR test results, subsequent NGS metagenomic sequencing was conducted.

Sequencing Results Comparison

A full-length sequence of mitochondrial genome of *N. fowleri* from the patient's CSF was obtained. Comparison of the complete sequence with the NCBI database showed that the mitochondrial complete sequences of *N. fowleri* from the United States (KX580902, KX580903), Japan (AP025248), and Pakistan (MZ461463) all reached a similarity of up to 99%.

Environmental Microbiology Laboratory Methods

Samples Procession

1. Types of samples

(1) Water: Collect 500–1,000 mL in a sterile container or sterile water sampling bag.

(2) Biofilms: Use a sterile wet cotton swab to scrape the biofilm from the surface, then collect a 100–300 mL sample of water flowing from the outlet or around the surface in the same pool.

2. Preservation and Delivery

The collected samples were preserved in room temperature and delivered to the reference laboratory in TCDC within 2 hours.

qRT-PCR for *N. fowleri*

Various methods were employed to extract nucleic acids from water and swab samples (biofilm). The process for water samples involved filtration through a 0.22-μm pore size polyethersulfone membrane (431118, Corning), followed by nucleic acid extraction with a DNeasy PowerWater Kit (Qiagen). Swab specimens underwent mixing by vortexing; the supernatant was then mixed with 6M guanidine thiocyanate (GIT) at a ratio of 1:1 and heated at 95°C for 30 minutes, followed by centrifugation at 13,000 rpm (15,871 ×*g*) for 1 minute. Nucleic acid extraction was conducted with an automatic nucleic acid extraction system (TANBead OptiPure Viral Auto Plate 665, Taiwan Dot).

A one-step qRT-PCR was used to amplify a 153-bp-long fragment of *N. fowleri* for this study. In brief, a 25-μL reaction included 5 μL of sample nucleic acid and 4X TaqMan Fast Virus 1-Step Master Mix (Thermo Fisher), 200 nM forward primer, 200 nM reverse primer, and

100 nM hydrolysis probe. The amplification program was perfomed on a LightCycler 480 (Roche), and reaction consisted of 53°C for 10 minutes and 95°C for 2 minutes, followed by 45 cycles of 95°C for 5 seconds and 60°C for 40 seconds, with a final cooling step of 40°C for 30 seconds.

Primer (*2*):

Naegl-F:GTGCTGAAACCTAGCTATTGTAACTCAGT

Naegl-R:CACTAGAAAAAGCAAACCTGAAAGG

Probe:

Naegl-P:HEX-ATAGCAATATATTCAGGGGAGCTGGGC-BHQ

References

1. Lin YC, Li MS, Chang JC, Huang SE, Tseng CP, Wu FT, et al. Application of next-generation sequencing in public health—detection of *Naegleria fowleri* by using metagenomic sequencing. Taiwan Epidemiology Bulletin. In press 2024.

2. Qvarnstrom Y, Visvesvara GS, Sriram R, da Silva AJ. Multiplex real-time PCR assay for simultaneous detection of *Acanthamoeba* spp., *Balamuthia mandrillaris*, and *Naegleria fowleri*. J Clin Microbiol. 2006;44:3589–95. PubMed https://doi.org/10.1128/JCM.00875-06
